# The effect of alcohol tax changes on retail prices: how do on-trade alcohol retailers pass through tax changes to consumers?

**DOI:** 10.1007/s10198-020-01261-1

**Published:** 2021-01-28

**Authors:** Luke B. Wilson, Robert Pryce, Colin Angus, Rosemary Hiscock, Alan Brennan, Duncan Gillespie

**Affiliations:** 1 School of Health and Related Research, University of Sheffield, 30 Regent Street, Sheffield, S1 4DA UK; 2 Tobacco Control Research Group, University of Bath, Claverton Down, Bath, BA2 7AY UK

**Keywords:** Alcohol, Alcohol excise duty, Tax pass-through, On-trade alcohol, Alcohol tax policy, Quantile analysis, I13, I18, H22

## Abstract

The effectiveness of alcohol duty increases relies on alcohol retailers passing the tax increase on to consumers. This study uses sales data from a market research company to investigate tax pass-through over 11 years for on-premise retailers in England and whether this varies across the price distribution, for different beverage categories and outlet types. Panel data quantile regression analysis is used to estimate the impact of 12 excise duty changes and 3 sales tax changes between 2007 and 2017 on prices. We use product-level quarterly panel data from for 777 alcoholic products. We undertake the regression at all outlets level separating products are analysed in seven broad beverage categories (Beer, Cider, RTDs, Spirits, Wine, Sparkling Wine, and Fortified Wine). We further test sensitivity by disaggregating outlets into seven outlet types. For all seven broad beverage categories, we find that there exists significant differences in tax pass-through across the price distribution. Retailers appear to “undershift” cheaper beverages (prices rise by less than the tax increase) and subsidise this loss in revenue with an “overshift” in the relatively more expensive products. Future modelling of tax change impacts on population subgroups could incorporate this evidence, and this is important because different socio-economic and drinker groups purchase alcohol at different points on the price distribution and hence are affected differently by tax changes. Governments could also potentially incorporate this evidence into future impact assessments.

**Supplementary Information:**

The online version contains supplementary material available at 10.1007/s10198-020-01261-1.

## Introduction

Alcohol consumption and its associated harms have been well documented and researched [1, 2], the result of which has led to excessive alcohol consumption becoming a great concern for policy makers and government officials. Nearly 4% of all deaths in England are related to alcohol annually and in 2017, there were 5,843 deaths from causes which are solely attributable to alcohol [3]. In order to reduce these figures policy makers have used a variety of alcohol control measures, the most common policy tool has been taxation in the form of excise duties.

Alcohol tax increases are used as a public health policy tool to reduce alcohol consumption and the negative externalities associated with alcohol. Tax and price increases have been shown to be effective in more than a hundred studies worldwide in reducing alcohol consumption and alcohol-related harms [4, 5]. More recently, Scotland introduced minimum unit pricing which sets a floor price for alcohol [6, 7]. This policy, however, has little impact on the on-trade (the on-trade can be defined as outlets like bars, restaurants, coffee shops, clubs, hotels, etc.) which is the focus of our study.

In an imperfectly competitive market, producers^1^ can use pricing strategies such that retailers or corporations can alter the size of the tax pass-through; thus, prices do not increase in proportion to the tax change (undershifting). On the other hand, retailers may in fact overshift the tax increase; therefore, prices are higher relative to the change in excise duty (overshifting) [8, 9]. Consequently, the effectiveness of alcohol tax policies depends on a variety of factors, including the consumer’s own- and cross-price elasticity of demand for alcohol [10], as well as the pricing strategies implemented by the retailer. Retailers or outlets can choose to absorb the excise duty increase such that it is not passed onto to consumers as an increase in retail price [11, 12]. This therefore undermines the effect of alcohol tax policy. Studies examining the UK cigarette market have shown that the tobacco industry “time” cigarette price changes at certain points in the financial year such that the price gap between the premium and ultra-low products appears larger, thus enabling smokers to downgrade to cheaper tobacco products [12, 13].

Previous empirical research on tax pass-through has come to various conclusions, evidence has shown that tax increases tend to be fully shifted onto consumers, or even overshifted on gasoline, factory made cigarettes, and alcohol, respectively [14–16]. On the other hand, researchers have found heterogeneous evidence of the under-/overshifting of tax and that this varies across the price distribution. Cawley and Frisvold [9] found that prices were lower than expected when a tax for sugar-sweetened beverages was introduced in the US, while Ally et al. [11] for alcohol and Hiscock et al. [12] for tobacco found evidence to suggest that retailers undershift on cheaper products and overshift on the more expensive varieties. With respect to alcohol, evidence in the USA finds that in the on-trade, beer prices are overshifted following an increase in sales tax of $0.07 in Alaska [16]. Additionally, Shrestha and Markowitz [17] provided estimates of beer tax pass-through estimates in the United States following on from the mergers of SABMiller and Coors in 2007 and Anheuser‐Busch and InBev in 2008. They use state‐level beer tax changes to suggest that a 10‐cent increase in beer taxes raises the retail prices of beer by roughly 17 cents [17]. In Belgium, researchers find evidence of heterogeneity in the pass-through of excise tax increases on the price of spirits. They too find evidence of overshifting and that tax was passed through quickly to the consumer during the first month of tax implementation. However, they find evidence of smaller tax shifts for some spirits in stores closer to the Luxembourg boarder [18].

This paper investigates the extent to which tax, in the form of excise duty and value-added sales tax, is passed through to consumers in the on-trade market in England. We extend the literature by decomposing our findings by beverage category and outlet type, and by examining pass-through across the price distribution. We find evidence to suggest that retailers implement a pricing strategy of undershifting tax on the cheapest products. This undermines the effectiveness of the public health intervention as heavier drinkers and heavy drinkers with lower incomes are at greater risk of harm from their drinking, and tend to purchase cheaper alcohol [19].

## Methods

### Data

In order to conduct our analysis, we use quarterly product-level panel data on prices of on-trade alcohol in England from the start of January 2007 to end of December 2017 (44 quarters). The source of the data is CGA Strategy, a UK-based market research company that specializes in location and brand market measurements. CGA manage the On-Premise Measurement Service which records retail price from a nationwide sample of on-trade outlets by product for a known dispense measure. Quarterly data are available on 777 individual products (also referred to as Stock Keeping Units) across eight regions of England.

The data covers prices from roughly 2000 on-premise locations in seven different outlet types: Hotel, Independent Pub, Managed Pub, Non-Managed Pubs, Proprietary Club, Restaurant, and Sports/Social Clubs. These different outlet types cover a variety of products across the price distribution. Within the data, the following information was available for each product: price, dispense measure at the point of sale (e.g. 568 ml), anonymous product number, Alcohol by Volume (ABV), five broad alcohol categories (beer, cider, Ready to Drinks (RTDs), spirits, and wine), and 23 narrow beverage categories. For this analysis, we use both the broad and narrow alcohol categories to focus on seven alcohol categories (beer, cider, RTDs, spirits, wine, sparkling wine, and fortified wine) to coincide with the alcohol excise duty rates that vary by these product types. Unit^2^ content for each product is calculated using the product size in litres multiplied by the recorded ABV. As well as the product information outlined above, CGA also record the volume of sales for each beverage. As a result, as well as knowing the price and characteristics of a particular product, we also know the sum of the total number of sales for each product over the course of our period of analysis (2007–2017) [20]. We can therefore use this information to weight the analysis by sales volume.

The unit of analysis is the combination of SKU, region, and outlet type. This gives 240,732 unique units of observation. The panel is unbalanced with an average observation per unit of 26.6 quarters (out of a possible 44). Over 25% of the sample has 44 observations.

### Taxation Changes between years 2006 and 2017

Our period of analysis covers 11 years of excise duty and value-added tax (VAT, UK sales tax charged as a percentage of price, also referred to as an ad valorem tax) changes across the various drink categories. Over this time, there have been 12 episodes of excise duty changes and three VAT changes. The exact dates for a particular change in excise duty or VAT are illustrated in Table 1. Table 2 provides a more detailed description to the changes in alcohol excise duty that has occurred over this time. All alcohol sold in the UK is subject to an ad valorem tax in the in the form of VAT. At the start of our analysis, VAT was 17.5% of the sales price. VAT was reduced from 17.5% to 15% on 1 December 2008 (quarter 9); it reverted back to 17.5% on 1 January 2010 (quarter 13) and increased again to 20% on 4 January 2011 (quarter 17).

**Table 1:** Duty and VAT changes in the UK

Budget date	Was there an excise duty change?	Change in VAT	Quarter (observed in the dataset)
26/03/06	–		–
26/03/07			1
17/03/08	**√**		6
01/12/08	**√**	17.5–15%	9
23/04/09	**√**		10
01/01/10		15–17.5%	13
29/03/10	**√**		14
04/01/11		17.5–20%	17
28/03/11	**√**		18
26/03/12	**√**		22
25/03/13	**√**		26
24/03/14	**√**		30
23/03/15	**√**		34
21/03/16	**√**		38
13/03/17	**√**		42

Illustrates the date a particular Duty, VAT, and Duty or VAT change took place in our period of analysis as well as its corresponding quarter in which we observe the change

**Table 2:** UK alcohol excise duty between January 2007 and December 2017

Tax event	Mar 2006	Mar 2007	Mar 2008	Dec 2008	Apr 2009	Mar 2010	Mar 2011	Mar 2012	Mar 2013	Mar 2014	Mar 2015	Mar 2016	Mar 2017
Panel a: excise tax for each beverage type over time													
Beer^a^	13.26	13.71	14.96	16.15	16.47	17.32	18.57	19.51	19.12	18.74	18.37	18.37	19.08
Strong beer^b^							23.21	24.39	27.21	23.99	23.85	23.85	24.77
Cider	25.61	26.48	28.90	31.21	31.83	36.01	35.87	37.68	39.66	39.66	38.87	38.87	40.38
Wine	172.17	177.99	194.28	209.82	214.02	225.00	241.23	253.39	266.72	273.31	273.31	277.84	288.65
Sparkling wine	220.54	227.99	248.85	268.75	274.13	288.20	308.99	324.56	341.63	350.07	350.07	355.87	369.72
Fortified wine	229.55	237.55	259.02	279.74	285.33	299.97	321.61	337.82	355.59	364.37	364.37	370.41	384.82
Spirits	19.56	19.56	21.35	22.20	22.64	23.80	25.52	26.81	28.22	28.22	27.66	27.66	28.74
RTDs	19.56	19.56	21.35	22.20	22.64	23.80	25.52	26.81	28.22	28.22	27.66	27.66	28.74
Panel B: change in excise tax from previous period (£ per hectolitre i.e. 100 L of beverage)^a^)													
Beer		0.45	1.25	1.19	0.32	0.85	1.25	0.94	-0.39	− 0.38	− 0.37	0.00	0.71
Strong beer							5.89	1.18	2.82	− 3.22	− 0.14	0.00	0.92
Cider		0.87	2.42	2.31	0.62	4.18	− 0.14	1.81	1.98	0.00	− 0.79	0.00	1.51
Wine		5.82	16.29	15.54	4.20	10.98	16.23	12.16	13.33	6.59	0.00	4.53	10.81
Sparkling wine		7.45	20.86	19.90	5.38	14.07	20.79	15.57	17.07	8.44	0.00	5.80	13.85
Fortified wine		8.00	21.47	20.72	5.59	14.64	21.64	16.21	17.77	8.78	0.00	6.04	14.41
Spirits		0.00	1.79	0.85	0.44	1.16	1.72	1.29	1.41	0.00	− 0.56	0.00	1.08
RTDs		0.00	1.79	0.85	0.44	1.16	1.72	1.29	1.41	0.00	− 0.56	0.00	1.08
Panel C:: percentage change in excise tax compared with previous period													
Beer		+ 3.39	+ 9.12	+ 7.95	+ 1.98	+ 5.16	+ 7.22	+ 5.06	− 2.00	− 1.99	− 1.97	0.00	+ 3.86
Strong beer							+ 34.01	+ 5.08	+ 11.56	− 11.83	− 0.53	0.00	+ 3.86
Cider		+ 3.40	+ 9.14	+ 7.99	+ 1.99	+ 13.13	− 0.39	+ 5.05	+ 5.26	0.00	− 1.99	0.00	+ 3.88
Wine		+ 3.88	+ 9.15	+ 8.00	+ 2.00	+ 5.03	+ 7.21	+ 5.04	+ 5.26	+ 2.47	0.00	+ 1.66	+ 3.89
Sparkling wine		+ 3.88	+ 9.15	+ 8.00	+ 2.00	+ 5.03	+ 7.21	+ 5.04	+ 5.26	+ 2.47	0.00	+ 1.66	+ 3.89
Fortified wine		+ 3.88	+ 9.15	+ 8.00	+ 2.00	+ 5.03	+ 7.21	+ 5.04	+ 5.26	+ 2.47	0.00	+ 1.66	+ 3.89
Spirits		0.00	+ 9.15	+ 3.98	+ 1.98	+ 5.12	+ 7.23	+ 5.05	+ 5.26	0.00	− 1.98	0.00	+ 3.90
RTDs		0.00	+ 9.15	+ 3.98	+ 1.98	+ 5.12	+ 7.23	+ 5.05	+ 5.26	0.00	− 1.98	0.00	+ 3.90

Cider, Wine, Sparkling Wine, and Fortified Wine are calculated as £ per hectolitre of product. We only observe lower strength cider (≤ 7.5% ABV) in the data, so cider is modelled as a single duty band. Similarly for wine, we only observe wine which is more than 5.5%, up to 15%, hence is modelled as a single duty. Sparkling wine modelled is greater than 8.5%. Spirits and RTDs is calculated as £ per litre of pure alcohol

^a^Beer duty is calculated £ per hectolitre per 1% ABV

^b^The UK introduced high- and low-strength duty bands in October 2011

In order to understand alcohol tax pass-through in a UK setting and interpret the results, it is important to understand how UK alcohol taxes are calculated and how they have evolved over time. UK excise tax duty on alcohol is determined by either the volume of pure alcohol (beer and spirits) or the volume of product (cider or all varieties of wine) depending on the beverage category. Beer is taxed using the volume of pure alcohol, and is taxed at £ per hectolitre (100L), per 1% of pure alcohol. Separate high- and low-strength beer excise duty rates were introduced in the October 2011 budget. Therefore, in our analysis, beer prices were calculated using one specific excise duty until October 2011 and calculated using separate duty rates above and below 7.5% thereafter.^3^ Alcohol duty for spirits is calculated as £ per litre of pure alcohol and RTDs are taxed the same as spirits. Cider and wine are both taxed using the volume of liquid in the bottle, i.e. duty is £ per hectolitre of product. Both beverage categories apply a different duty depending on the ABV %. Duty on wine varies by the strength and separates four categories of wine (≤ 15%, more than 15%, sparkling wine, and fortified wine).

The UK government introduced an “alcohol duty escalator” in March 2008 (quarter 6), which implemented an initial increase in alcohol duty of 6% as well as an annual 2% increase in the duty above inflation. The duty escalator was introduced as a measure to address the rising affordability of alcohol. The duty escalator was stopped in March 2013 (quarter 26) for beer and in March 2014 (quarter 30) for all other categories of alcohol, meaning that tax on products fell in real terms since.

### Calculating the expected price per unit given the tax change

One of the key variables of interest in our study is the expected price per unit $E\left[{\mathrm{Price}}_{\mathrm{it}}\right]$. This is the price per unit in pence for each SKU *i* assuming that there is full tax pass-through at the time of the duty change *t*. We calculate $E\left[{\mathrm{Price}}_{\mathrm{it}}\right]$ for each year and quarter time period (e.g. 2009q2) that the SKU is observed in the data.

The equation for expected price per unit is given in Eq. 1 for SKU *i* at time *t*:

1 $$\begin{array}{*{20}c} {E\left[ {{\text{Price}}_{{{\text{it}}}} } \right] = \left( {\left( {\frac{{{\text{Price}}_{{\text{it = 1}}} - {\text{Duty}}_{{\text{it = 1}}} }}{{1 + {\text{VAT}}_{t = 1} }} \times {\text{RPI}}_{t} } \right) + {\text{Duty}}_{{{\text{it}}}} } \right) \times \left( {1 + {\text{VAT}}_{t} } \right)} \\ \end{array} .$$

In order to calculate the expected price, $\mathrm{E}\left[{\mathrm{Price}}_{\mathrm{it}}\right]$ takes the value of the observed price in the first time period it appears in the data. To construct the evolution of $\mathrm{E}\left[{\mathrm{Price}}_{\mathrm{it}}\right]$ over time, we remove the amount of excise duty and VAT that would have been due at time *t* = *1*, this leaves only net revenue. Net revenue is the money the “producer” retains from its sales once all alcohol excise tax and VAT have been paid. This forms a “baseline” price, which is then inflated to real terms using the Retail Price Index (RPI).^4^ This inflated baseline price is then updated over the course of the time frame in the data to reflect the incremental change in excise duty and VAT in each following time period. By definition, expected price is equal to observed price the first time the product is observed in the data. Similarly, if a product’s price increases exactly in line with excise duty, VAT, and inflation, then its expected price is equal to observed price.

The expected price presented in this paper is inflated using RPI rather than the consumer price index (CPI) or the Harmonised Index of Consumer Prices (CPIH). While the latter has been used in previous pass-through literature, [12] we use RPI as this is used to set the path for most excise duty rates in the UK as described in the most recent UK budget “Duty rates on beer, most cider and spirits will be frozen. Duty on most wine and higher strength sparkling cider will rise by RPI inflation…” [21].

### Quantile Regression strategy to estimate tax pass-through across the price range

We exploit the panel nature of the price data and adopt a quantile regression approach using the *RQPD* package in R developed by Koenker [22]. Rather than focusing only on the predicted mean of the dependent variable, as in classical linear regression, quantile regression focuses on quantiles which refer to defined points in the distribution. For example, the 0.50 quantile is the median and 0.05 is the 5th percentile of the distribution. This allows for the flexibility for modelling the entire distribution of prices. This methodology provides a framework for investigating differential tax pass-through for price points across the entire price distribution. Moreover, by using this approach, it allows our calculated expected price of each product to be included as an independent variable.

The basic version of our model is as follows:

2 $$\begin{array}{*{20}c} {{\text{ObservedPrice}}_{{{\text{it}}}} = \beta_{0} + \beta_{1,\theta } {\text{ExpectedPrice}}_{{{\text{it}}}} + \varepsilon_{{{\text{it}},\theta }} } \\ \end{array} ,$$

where ${\text{ObservedPrice}}_{{{\text{it}}}}$ is the observed price per unit of product *i* at a specific time $t$ and ${\text{ExpectedPrice}}_{{{\text{it}}}}$ is the price per unit calculated assuming a full tax pass-through.

We consider 11 quantiles (0.05, 0.15, 0.25, 0.35, 0.45, 0.5, 0.55, 0.65, 0.75, 0.85, and 0.95) including the median *θ* = 0.50. We run the model for each beverage category separately.

If tax changes (either duty or VAT changes) are fully passed onto consumers across the price distribution then, for all quantiles, the estimated *β* coefficient of a given product in on-trade location should equal 1. If *β* is less than 1, this is an example of undershifting and the producer is losing some revenue and bears some of the burden of the tax change. If *β* is great than 1, this represents overshifting and the consumer is paying more than the 100% tax pass-through expected price given the tax change, and the producer is gaining additional revenue.

#### Descriptive statistics on quantiles of price

Table 3 illustrates the absolute price per unit for the upper bound of each quantile band (*θ*) as well as the proportion of on-trade sales for each beverage type that occurs in each band of the price distribution. The price distribution refers to the range of different unit prices paid for all products in each of the seven beverage categories. We also present the overall number of products in each specific beverage category. The range in price per unit for each beverage type varies substantially. Beer and cider have the smallest difference in price, as well a similar price per unit in each quantile band across the distribution, while on the other hand the unit prices of spirits and all of the wine varieties are much more dispersed. For comparison, for beers at the lowest end of the price distribution,^5^ the price per unit (*θ* = 0.05) is 90.44p and at the highest end (*θ* = 0.95) is 217.75p. While on the other hand, for spirits, the cheapest products are 136.47p per unit and the most expensive are 530.91p and for sparkling wines *θ* = 0.05 is 159.57p per unit and *θ* = 0.95 1472.58p per unit. An explanation for such large differences in the unit price of sparkling wine is that this category comprises of usually cheaper sparkling wines, such as Cava and Prosecco as well as traditionally more expensive Champagne.

**Table 3:** Quantiles of prices (pence per unit) and volume of sales

	Beer		Cider		RTDs		Spirits		Wine		Sparkling		Fortified wine	
	Price^a^	% Sold^b^	Price	% Sold	Price	% Sold	Price	% Sold	Price	% Sold	Price	% Sold	Price	% Sold
0.9 5 > *θ*	NA	1.13	NA	0.12	NA	0.48	NA	0.80	NA	8.51	NA	0.21	NA	3.43
0.85 < *θ* ≤ 0.95	217.75	2.41	234.50	1.03	263.38	1.78	530.91	3.48	274.87	18.65	1472.58	2.15	501.56	10.49
0.75 < *θ* ≤ 0.85	181.82	2.04	196.00	1.67	234.86	6.77	369.03	7.82	187.63	14.03	802.44	1.19	374.27	12.26
0.65 < *θ* ≤ 0.75	155.75	6.64	172.29	2.47	223.27	14.66	309.59	13.68	157.36	12.49	667.89	7.36	328.63	12.42
0.55 < *θ* ≤ 0.65	141.08	14.50	154.95	5.63	213.09	20.37	278.57	12.61	139.56	9.06	580.01	11.93	295.87	13.10
0.50 < *θ* ≤ 0.55	131.00	6.00	143.80	1.52	202.43	6.03	254.11	9.19	127.23	2.67	512.31	14.58	273.67	5.86
0.45 < *θ* ≤ 0.50	126.68	11.72	139.48	1.97	198.60	5.84	244.36	7.07	123.27	4.23	484.89	4.02	263.16	3.38
0.35 < *θ* ≤ 0.45	123.09	13.85	135.51	5.99	194.77	13.51	233.73	12.68	118.61	6.97	443.18	14.79	250.62	12.33
0.25 < *θ* ≤ 0.35	116.61	22.88	126.29	22.24	187.93	10.89	214.50	10.51	109.44	8.87	377.69	4.95	228.15	10.15
0.15 < *θ* ≤ 0.25	110.04	12.01	118.23	23.83	181.43	9.17	193.88	9.94	100.74	8.48	328.79	9.63	206.72	7.73
0.05 < *θ* ≤ 0.15	102.34	6.03	108.95	31.44	170.49	9.12	171.46	9.00	90.34	4.63	281.21	18.78	182.61	7.92
*θ* ≤ 0.05	90.44	0.79	93.33	2.10	155.68	1.38	138.47	3.22	75.15	1.41	159.57	10.40	147.46	0.92

^a^Price per unit refers to the upper bound of each quantile band (*θ*)

^b^Volume sold is the percentage sold in this category at this price band as a percentage of all alcohol sold in this category Price distribution for a specific product class captures the unit prices of all products falling within the category NA refers to not applicable. Volume Sold refers to the % sold in each price band; due to rounding, total sales may not sum to 100%

## Results

### Quantile regression results

**Fig. 1:**
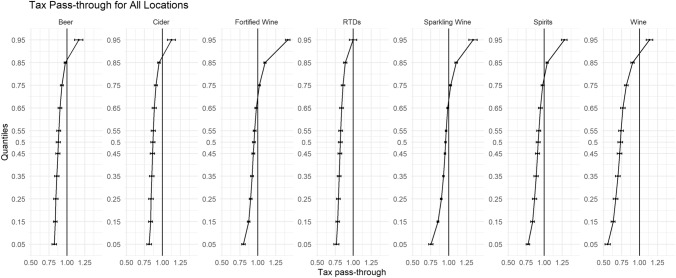
Tax pass-through for all locations

Figure 1 displays the beverage-specific tax pass-through estimates for all on-premise outlet types (see appendix for the regression parameters). Between the 5% and 50% quantiles, there is evidence to suggest undershifting (i.e. pass-through less than 1) for all beverage varieties. Furthermore, at the lowest quantile (*θ* = 0.05), undershifting is more pronounced for wine 0.55 (0.50, 0.60), sparkling wine 0.75 (0.72, 0.78), and RTDs 0.76 (0.74, 0.78).

For all beverage categories, except RTDs our estimates suggest that there is significant overshifting (i.e. pass-through greater than 1) for products at the highest end of the price distribution. This overshifting of tax is evident for all beverages in the top quantile (*θ* = 0.95). This is in contrast to the previous literature on alcohol tax pass-through using quantile panel methods who find evidence of overshifting from the *θ* = 0.15 quantile onwards [11]. This portrays the sense that on-trade retailers find it significantly harder to pass on excise tax rises onto consumers than the off-trade.

Evidence of undershifting is apparent for the cheapest 65% of all products and extends further along the price distribution. Our results suggest that tax rises lead to price increases in the cheapest 5% of products which are 10% lower than full pass-through for beer and cider, 18% lower for spirits and RTDs, and 45% lower for wines. For all beverage categories, the magnitude of overshifting increases for the products at the higher end of the price distribution.

For the most expensive products, changes in tax is passed-though above the rate of duty such that the prices for wine are 25% higher than their expected price post duty increase. Furthermore, for beer and cider, prices are 30% and 23% higher than full pass-through. Figure 1 illustrates the full extent of magnitude of tax pass-through for all beverage categories in all outlet types.

### Quantile regression results: sub-investigation of variation by on-trade outlet type

**Fig. 2:**
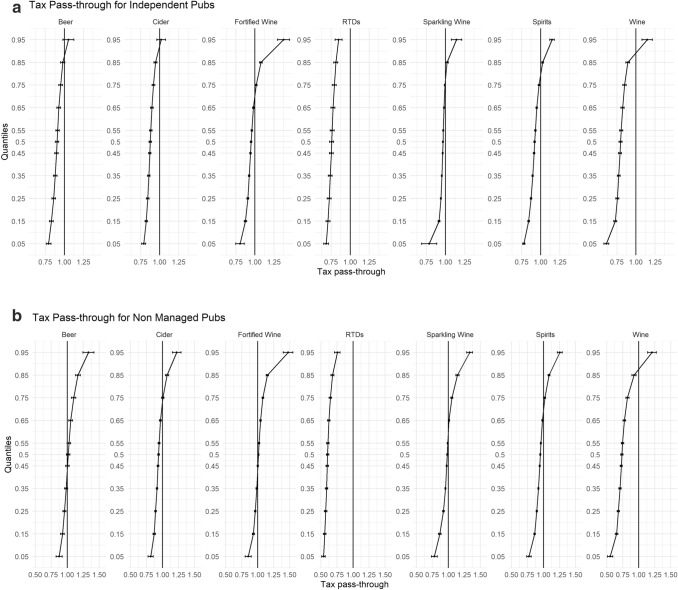

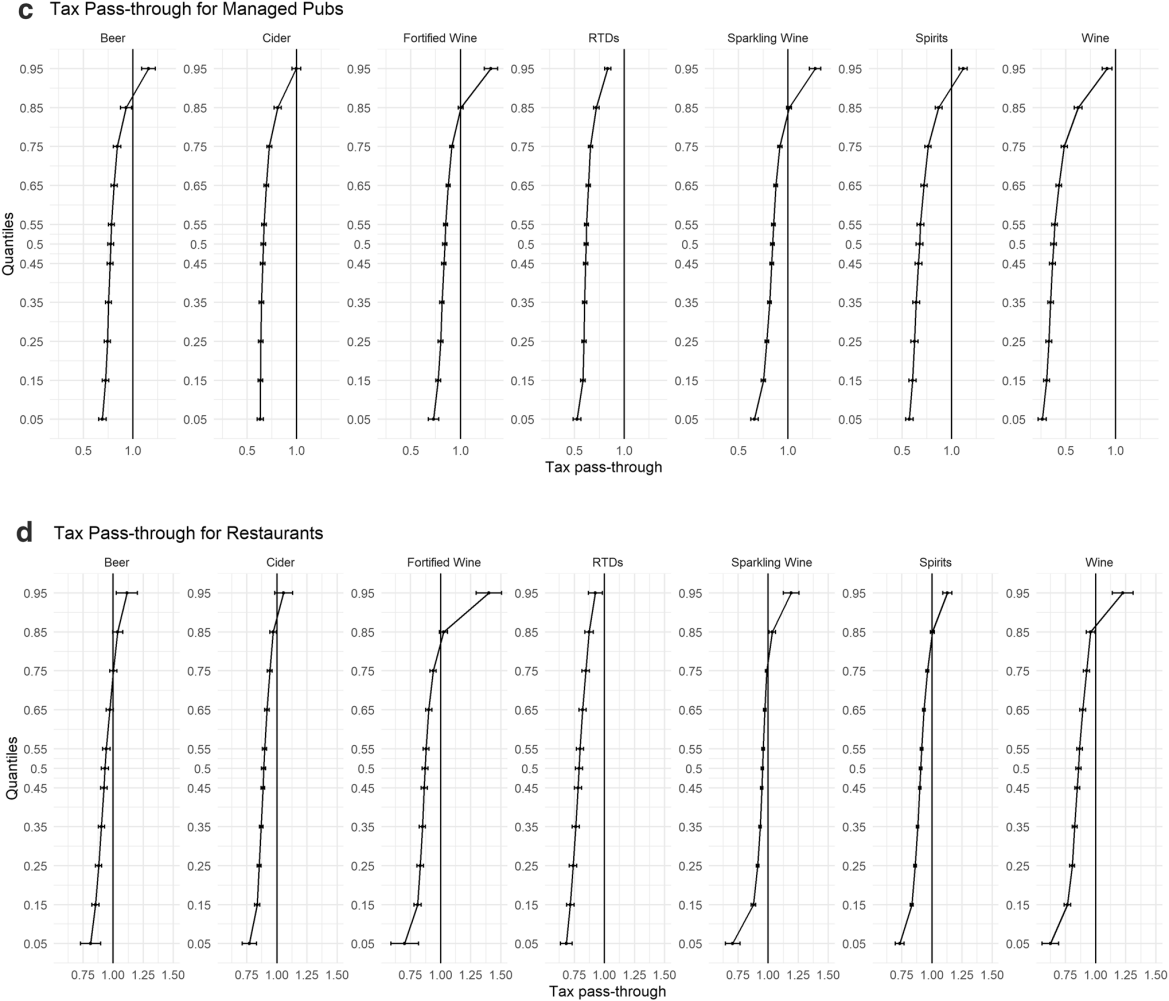

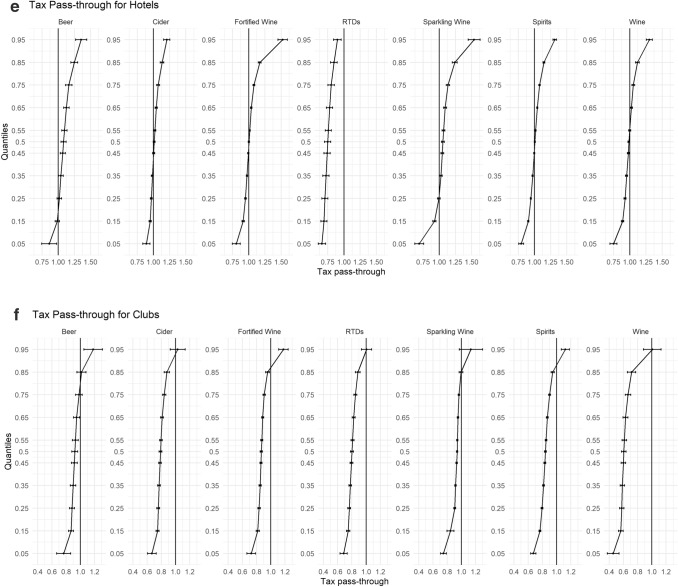

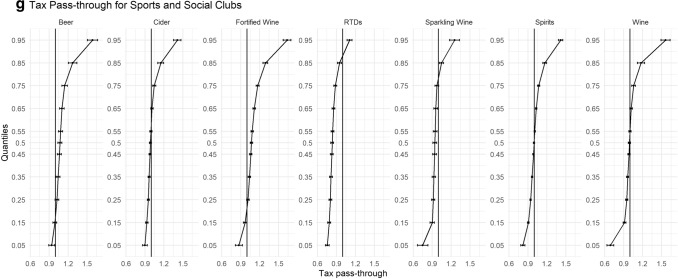
**a** Tax pass-through for independent pubs. **b** Tax pass-through for non-managed pubs. **c** Tax pass-through for managed pubs. **d** Tax pass-through for restaurants. **e** Tax pass-through for hotels. **f** Tax pass-through for proprietary clubs. **g** Tax pass-through for sports and social clubs

In Fig. 2a–g, we disaggregate our findings further by stratifying our sample into the different on-trade locations (Hotel, Independent Pub, Managed Pub, Non-Managed Pubs, Proprietary Club, Restaurants, and Sports/Social Club). Each of these on-premise locations have their own unique characteristics and revenue streams that could lead to heterogeneous levels of tax pass-through.

One of the most striking results from this analysis is the difference in pass-through among the three different types of pubs. While each different outlet illustrates undershifting in their cheapest products and overshifting in their more expensive, which are in agreement with our main results, what is surprising is the extent of undershifting in Managed Pubs for all beverage categories. In Managed Pubs, there is statistically significant evidence to suggest undershifting of tax across the price distribution until the 85% quantile for each beverage category, and evidence to suggest that RTDs and Wine are undershifted across the entire price distribution. In comparison, while undershifting for the relatively cheaper products is still occurring in Non-Managed pubs and Independent pubs, this only evident until the 35% and 75% quantiles, respectively, for most beverage categories.

#### Weighted analysis of tax pass-through sales volume

In order to examine the proportion of the total amount of sales affected by under- and overshifting, we use on-trade sales data for each product. Data are again supplied by CGA. These sales data capture the percentage share of the market that specific product has for our period of analysis. Thus, we can calculate the corresponding percentage of the market share for the corresponding quantile band for each of the seven beverage categories, both for the entire market and for each on-premise location. As a result, we are essentially applying the sales weights to the analysis but after the panel data quantile regression results. The percentage sold in each quantile band for the entire on-trade market are summarised in the respective “% Sold” columns of Table 3 and are depicted graphically in the supplementary materials.^6^

Linking the calculated rates of pass-through together with the volume of sales that are listed in Table 3, we find that roughly 67%^7^ of beer sales are undershifted and sold for less than 127p per unit. This is similar for fortified wines in which 17% of sales are sold below the expected price at less than 207p per unit. When linked together with their sales data, these two products have the lowest prevalence of undershifting within their specific beverage category. On the other hand, in terms of sales, 90% of cider is sold below the *θ* = 0.75 quantile band. This indicates that within the price distribution, ciders sold in the 75% quantile and below are sold below their expected price and account for 86% of the total amount of cider sold. Similarly, for RTDs we find that they are undershifted across all outlets up to and including the 85% quantile. This accounts for 97% of the market for RTDs.

Furthermore, for spirits and wine, when we link tax pass-through to their volume of sales, we find that undershifting occurs for 73% of all wine, 77% of all sparkling wine, and 88% of all spirits sold in each of these categories across all on-trade locations. By linking the volume of sales data to our quantile analysis, we are able to observe not only the prevalence of tax pass-through across the price distribution and its magnitude in each quantile but also to observe its ubiquity across all on-trade alcohol sales.

## Discussion

This paper uses quantile regression methods to estimate the level of tax pass-through in the on-trade market. For the first time, we analyse on-trade pass-through across beverage categories and outlet types, as well as across the price distribution within a beverage outlet category. Our results show substantial variation in pass-through. Specifically, we observe a clear difference in pass-through for cheap versus expensive alcohol, with the cheapest alcohol categories being undershifted most and the more expensive overshifted. The magnitude of pass-through ranges from 55% for the lowest priced wines to 142% for the most expensive fortified wines. The median pass-through for each of the seven beverage categories is examined is below 100%, i.e. all types of alcohol are on average undershifted. When looking at outlet categories 4 of the seven categories have a median pass-through below 100% for all types of alcohol-independent pubs, managed pubs, restaurants, and proprietary clubs.

Apart from Kenkel et al. [16], who examined tax pass-through of malted products in Alaska in the on-trade and off-trade, we are not aware of any other studies showing on-trade alcohol tax pass-through. However, there are numerous that examine tax pass-through for off-trade. Our study produces similar findings to Ally et al. [11], who in the off-trade found evidence of undershifting at the lowest quartiles, but found overshifting at the highest quantiles. However, in contrast to that of Ally et al. [11] we find that within the on-trade, undershifting occurs in almost all of the quantiles across the price distribution, with retailers only overshifting in the highest quantiles [11]. Findings in the literature do vary by country and two studies show an off-trade pass-through in US and Belgium with overshifting. This suggests that our results may not be generalisable beyond the UK, indeed the CGA data are only for the eight regions of England. A possible explanation as to why we find highly prevalent evidence of undershifting of tax in the on-trade, especially when evidence in the off-trade shows evidence of overshifting to various magnitudes [16–18], could be due to the pressure to not further widen the price differential between off-trade and on-trade, which has widened recently with the off-trade being considerably cheaper. This is important to take into consideration as evidence has shown that consumers swap to cheaper products [23].

However, the work is not without limitations. One limitation in the data is the classification of a stock keeping unit (SKU) in the wine product classes. In the on-trade, wine is unique in that wine brands are not as ubiquitous as other alcohol types, and that customers are rarely loyal to a particular wine brand. Instead, customers are more likely to choose wine based on grape type and country of origin, as well as other factors, including price. As a consequence of this weak brand loyalty, the exact same SKU of wine is rarely observed at every time point. To deal with this, CGA first attempt to collect price data on a specific SKU where enough price observations exist. Where this is not possible, a synthetic SKU is created based upon a wine’s country of origin and grape variety, for example, French Cabernet Sauvignon. This methodological approach creates the limitation that we are unable to fully disentangle the observed price in wine, as fluctuations in price can be an actual change in price of the brand variant at the point of sale or a change in products stocked in each on-trade location.

We have not examined the supply side cost base of the on-trade premises because of a lack of available data. However, potential avenues for further research on explanatory factors related to the premises cost base include the following: obtaining data on average business rates charged to the 7 types of premises, by region, over time included in this study; obtaining data on the costs of goods and services supplied to the on-trade premises concerned, through some commercial database or modelling exercise. Both of these would demand substantial investment in financial accounting data that are not currently readily available.

We have not examined changes in the extent of undershifting over time because our quantile regression essentially averages the changes in tax and process over the 2007–2017 period. New analytical approaches would be needed to examine whether there is an acceleration in the extent of undershifting over time, or whether the changes in the numbers of pubs and bars that are open over time, or indeed other factors, such as licensing hours and other contextual conditions, are causing changes in undershifting patterns.

Theory as to why undershift occurs differentially for different products—Outlets may be able to absorb some of the tax increases directly applying differential strategies to different products, i.e. subsidising, e.g. passing beer tax increases onto the price of wine or through increases in the price of non-alcohol-related products, such as food or soft drinks. To answer this question would require obtaining data on the sales of and process of other products which on-trade venues earn revenue from including, food, soft drinks, gaming activities, etc.

The next steps in our own research programme include applying these results on undershifting, alongside those for the off-trade from Ally et al. [11] and new emerging work on tobacco tax pass-through to have a co-ordinated analysis of the impact of both alcohol and tobacco tax policies on purchasing, consumption, and harm.

Our results indicate that tax increases lead to increases in the price of alcohol across the price distribution, therefore support the extensive evidence on the effectiveness of duty increases on reducing alcohol consumption. However, the pricing strategy of undershifting tax across the price distribution on the cheapest products undertaken by retailers undermines the effectiveness of the public health intervention as heavier drinkers and heavy drinkers with lower incomes are at greater risk of harm from their drinking and tend to purchase cheaper alcohol [19]. Therefore, additional measures may be required to ensure that policies are well targeted.

## Supplementary Information

Below is the link to the electronic supplementary material.

Supplementary file1 (DOCX 1459 KB)

## Data Availability

Data are provided by CGA Ltd and are not sharable due to commercial sensitivity.

## Footnotes

1 The term “producer” is used in this paper in the economics sense, reflecting the supply-side of the market. One can think of this as the supply chain from manufacturer to retailer.

2 We use the UK definition of an alcohol unit which is 1 unit is 8 g/10 ml of pure alcohol. A unit is the equivalent of one 25 ml measure of spirit. Additionally, a pint (568 ml) of 5% beer is 2.8 units.

3 In our data, there were no beers sold with an ABV < 2.8%; we therefore do not include their duty rates.

4 RPI is a measure of inflation published monthly by the Office for National Statistics (ONS). It measures the change in the cost of a representative sample of retail goods and services. The UK Treasury uses RPI for various index linked tax rises.

5 An average pint of beer is roughly 2.5 units so at this price per unit, the price of a pint would be £2.26. A pint of beer which is 2 units this would equate to £1.80.

6 Tabulated estimates of the percentage sold in each quantile band as well as their corresponding price per unit by beverage category and for each individual on-trade location can be provided by the authors upon request.

7 This is the sum of all values in “% sold” for the beverage type in which the quantile band was undershifted (β < 1). This is consistent for the remaining percentages relating to the percentage sold of other beverages listed in this section.

## Code availability

Analysis was conducted in Stata 15. Code can be made available to accredited researchers upon request.
