# Supplementary material for: The effect of alcohol tax changes on retail prices: how do on-trade alcohol retailers pass through tax changes to consumers?
Source: Eur J Health Econ. 2021 Jan 28;22(3):381–92. doi: 10.1007/s10198-020-01261-1 (PMC7954722; doi:10.1007/s10198-020-01261-1)
Supplement: Supplementary file 1 — Supplementary file1 (DOCX 1459 KB) [file 10198_2020_1261_MOESM1_ESM.docx]

**The effect of alcohol tax changes on retail prices. How do on-trade alcohol retailers pass-through tax changes to consumers?**

**Supplementary Document**

| Table S1: Calculation of duty paid and expected price of seven selected exemplar products | | | | | | | | | | | | | | | | | |
| --- | --- | --- | --- | --- | --- | --- | --- | --- | --- | --- | --- | --- | --- | --- | --- | --- | --- |
|  |  |  |  | Date | Jan-07 | Mar-07 | Mar-08 | **Dec-08** | Apr-09 | **Mar-10** | **Mar-11** | Mar-12 | Mar-13 | Mar-14 | Mar-15 | Mar-16 | Mar-17 |
|  |  |  |  | RPI | 1 | 1.01 | 1.06 | 1.04 | 1.06 | 1.1 | 1.16 | 1.19 | 1.23 | 1.26 | 1.27 | 1.29 | 1.34 |
| Product | | | |  |  |  |  |  |  |  |  |  |  |  |  |  |  |
| Category | Volume (ml) | ABV^1^ | Units |  |  |  |  |  |  |  |  |  |  |  |  |  |  |
| Beer | 568 | 4% | 2.27 | Duty | 13.2 | 13.64 | 14.96 | 16.29 | 16.29 | 17.17 | 18.49 | 19.37 | 18.93 | 18.93 | 18.49 | 18.49 | 18.93 |
|  |  |  |  | E[P]^2^ | 97 | 99 | 105 | 103 | 104 | 112 | 122 | 127 | 131 | 134 | 135 | 136 | 142 |
| Cider | 568 | 4.50% | 2.56 | Duty | 5.87 | 5.87 | 6.26 | 7.04 | 7.04 | 7.82 | 7.82 | 8.22 | 9 | 9 | 8.61 | 8.61 | 9 |
|  |  |  |  | E[P] | 117 | 119 | 125 | 121 | 123 | 132 | 141 | 146 | 152 | 156 | 157 | 159 | 165 |
| RTD | 275 | 5.50% | 1.51 | Duty | 19.83 | 19.83 | 21.16 | 22.48 | 22.48 | 23.8 | 25.79 | 27.11 | 28.43 | 28.43 | 27.77 | 27.77 | 28.43 |
|  |  |  |  | E[P] | 162 | 165 | 173 | 168 | 171 | 183 | 200 | 208 | 216 | 22 | 223 | 226 | 235 |
| Spirits | 25 | 38% | 0.95 | Duty | 20 | 20 | 21.05 | 22.11 | 23.16 | 24.21 | 25.26 | 26.32 | 28.42 | 28.42 | 27.37 | 27.37 | 28.32 |
|  |  |  |  | E[P] | 192 | 195 | 204 | 197 | 202 | 234 | 242 | 253 | 259 | 259 | 260 | 264 | 275 |
| Wine | 175 | 12.50% | 2.19 | Duty | 13.71 | 14.17 | 15.54 | 16.91 | 16.91 | 17.83 | 19.2 | 20.11 | 21.49 | 21.94 | 21.94 | 22.4 | 23.31 |
|  |  |  |  | E[P] | 83 | 85 | 90 | 88 | 89 | 96 | 105 | 110 | 115 | 119 | 120 | 123 | 128 |
| Sparkling Wine | 750 | 12.50% | 9.38 | Duty | 17.6 | 18.24 | 19.95 | 21.55 | 21.97 | 23.04 | 24.75 | 25.92 | 27.31 | 28.05 | 28.05 | 28.48 | 29.55 |
|  |  |  |  | E[P] | 284 | 290 | 304 | 294 | 299 | 320 | 346 | 358 | 371 | 382 | 386 | 392 | 407 |
| Fortified Wine | 50 | 17.50% | 0.88 | Duty | 12.57 | 13.71 | 14.86 | 16 | 16 | 17.14 | 18.29 | 19.43 | 20.57 | 20.57 | 20.57 | 21.71 | 21.71 |
|  |  |  |  | E[P] | 109 | 112 | 118 | 114 | 116 | 125 | 136 | 142 | 148 | 152 | 153 | 157 | 163 |
| **Notes:** ^1^ABV is Alcohol by Volume. ^2^E[P] is the expected price per unit of that particular product at that particular time in pence. The individual products depicted in this table were selected at random. They are used to illustrate the evolution of duty and expected price per unit over time as well as illustrate how prices change with respect to changes in VAT and changes in inflation (RPI) over time. The text in bold refers to whether there was a change in VAT in that year. | | | | | | | | | | | | | | | | | |

Figure S1: Comparison of actual observed inflation adjusted price per unit of alcohol versus predicted expected price if tax pass-through were always 100%


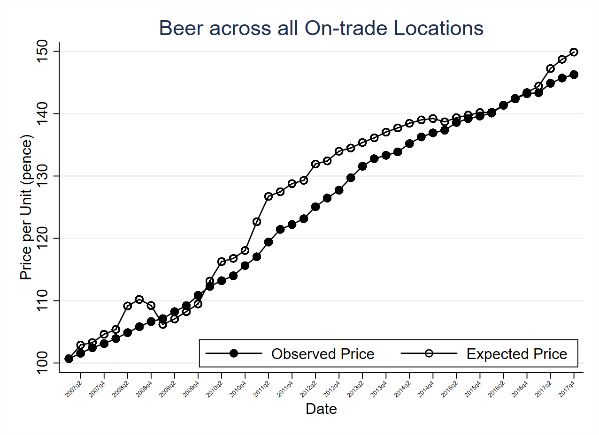

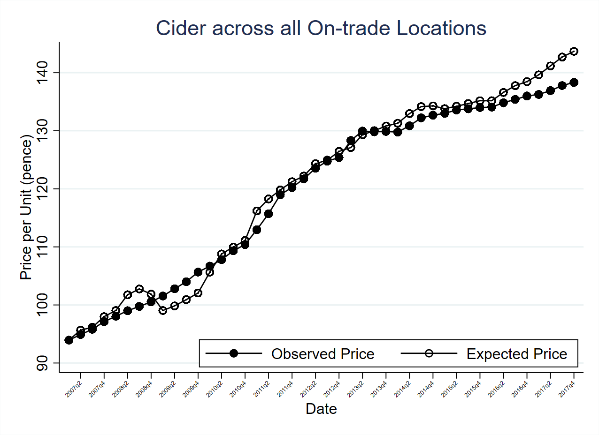

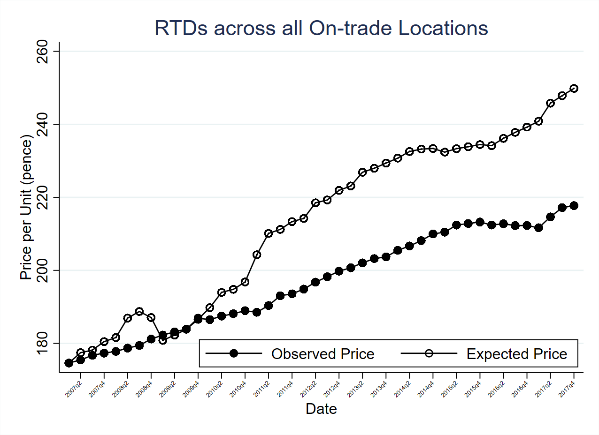

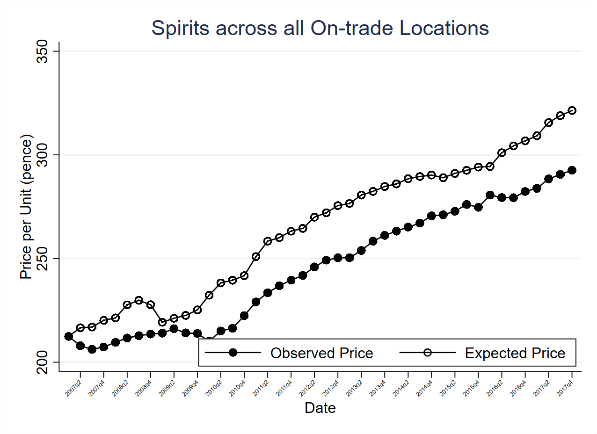

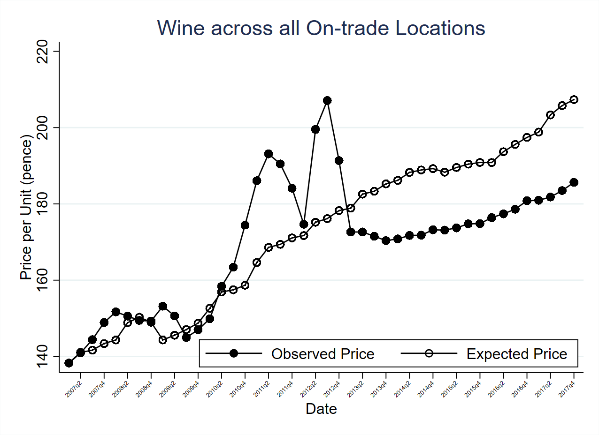

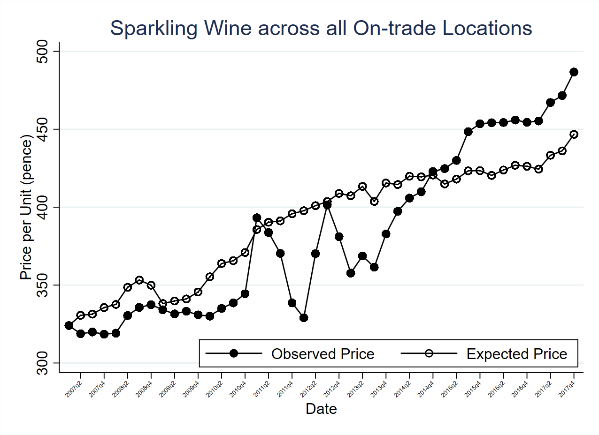

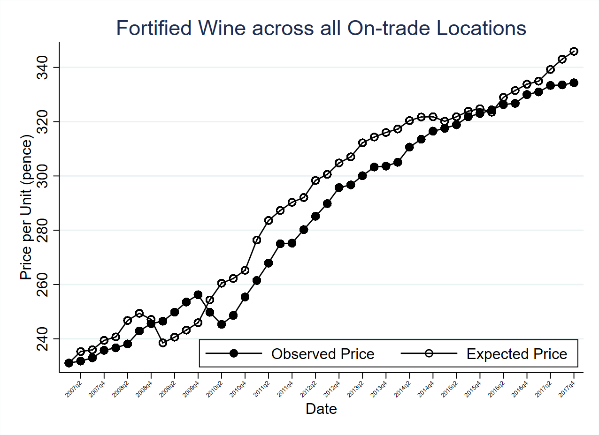


| Table S1: Point estimates from Figure 2 | | | | | | | |
| --- | --- | --- | --- | --- | --- | --- | --- |
| Quantile | Beer | Cider | RTDs | Spirits | Wine | Sparkling Wine | Fortified Wine |
| 0.05 | 0.824 | 0.816 | 0.765 | 0.774 | 0.556 | 0.752 | 0.799 |
|  | (0.019) | (0.019) | (0.020) | (0.015) | (0.021) | (0.016) | (0.014) |
| 0.15 | 0.838 | 0.836 | 0.782 | 0.839 | 0.631 | 0.850 | 0.872 |
|  | (0.014) | (0.015) | (0.014) | (0.015) | (0.016) | (0.007) | (0.009) |
| 0.25 | 0.846 | 0.841 | 0.792 | 0.866 | 0.666 | 0.897 | 0.899 |
|  | (0.016) | (0.017) | (0.014) | (0.016) | (0.018) | (0.007) | (0.010) |
| 0.35 | 0.859 | 0.852 | 0.803 | 0.889 | 0.697 | 0.927 | 0.919 |
|  | (0.016) | (0.017) | (0.014) | (0.016) | (0.018) | (0.006) | (0.010) |
| 0.45 | 0.873 | 0.862 | 0.813 | 0.908 | 0.721 | 0.947 | 0.937 |
|  | (0.016) | (0.016) | (0.014) | (0.015) | (0.018) | (0.006) | (0.010) |
| 0.50 | 0.880 | 0.868 | 0.819 | 0.917 | 0.732 | 0.956 | 0.946 |
|  | (0.015) | (0.016) | (0.014) | (0.015) | (0.017) | (0.006) | (0.010) |
| 0.55 | 0.886 | 0.873 | 0.824 | 0.925 | 0.741 | 0.964 | 0.955 |
|  | (0.015) | (0.016) | (0.014) | (0.015) | (0.017) | (0.006) | (0.010) |
| 0.65 | 0.903 | 0.886 | 0.837 | 0.947 | 0.767 | 0.986 | 0.979 |
|  | (0.014) | (0.015) | (0.013) | (0.014) | (0.016) | (0.007) | (0.009) |
| 0.75 | 0.929 | 0.909 | 0.857 | 0.980 | 0.814 | 1.026 | 1.020 |
|  | (0.012) | (0.012) | (0.013) | (0.012) | (0.014) | (0.008) | (0.008) |
| 0.85 | 0.977 | 0.953 | 0.888 | 1.043 | 0.901 | 1.103 | 1.100 |
|  | (0.008) | (0.009) | (0.013) | (0.008) | (0.012) | (0.010) | (0.008) |
| 0.95 | 1.164 | 1.126 | 0.997 | 1.281 | 1.137 | 1.339 | 1.416 |
|  | (0.033) | (0.032) | (0.030) | (0.022) | (0.025) | (0.027) | (0.016) |

Additional Results

**Figure S2:** Model Estimates using Consumer Price Index including owner occupiers’ housing costs (CPIH) instead of the Retail Prices Index (RPI)

Figure S2a: All Outlet Locations (CPIH)


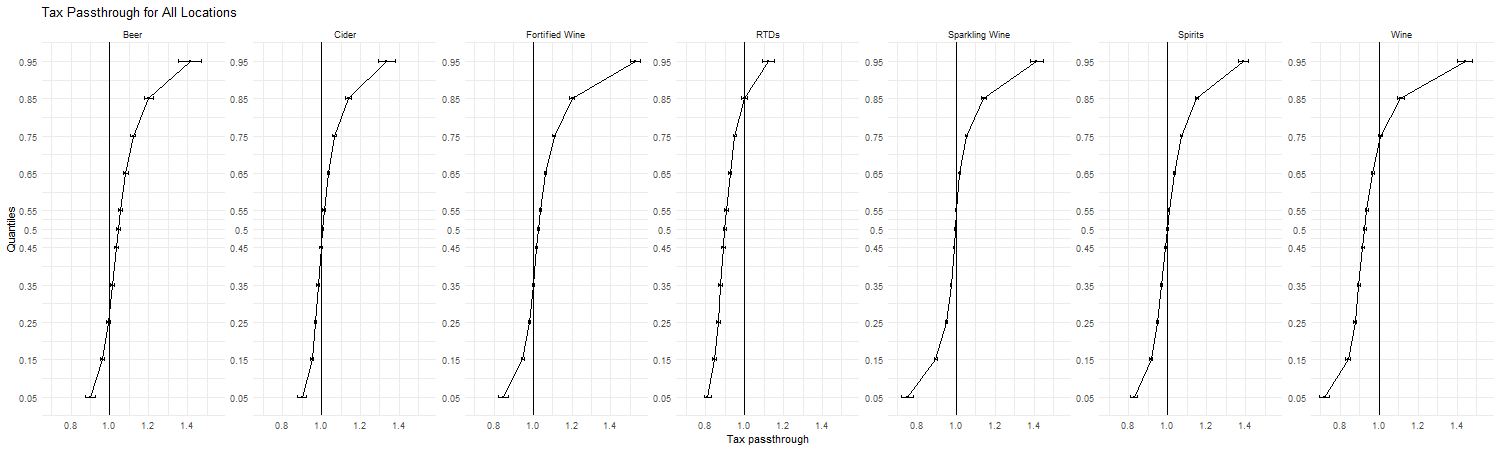


Figure S2b: Independent Pubs (CPIH)


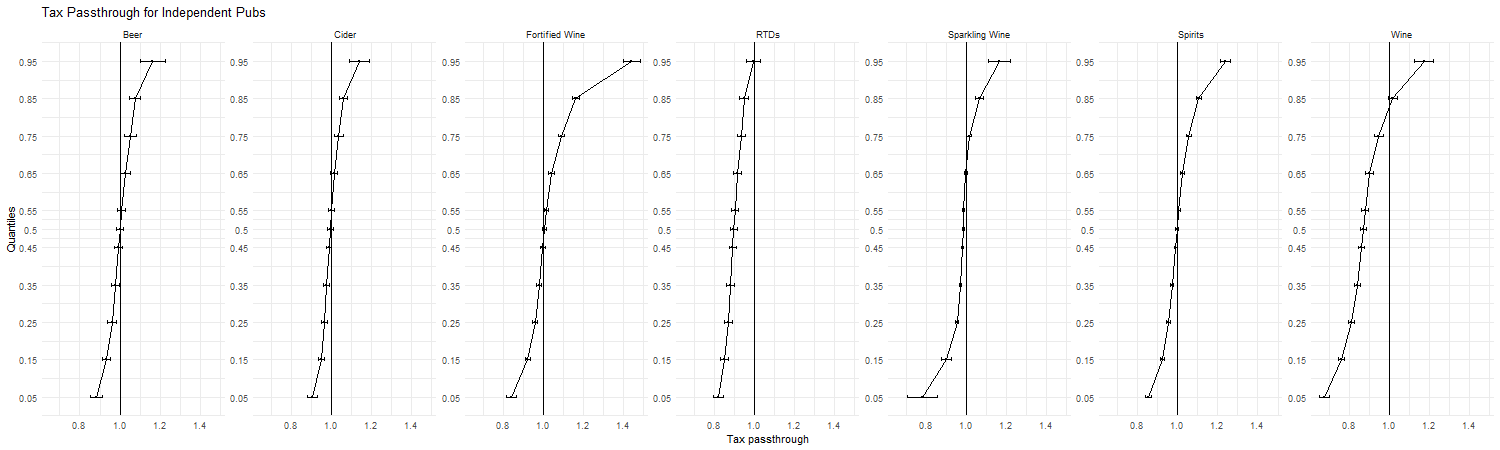


Figure S2c: Non-Managed Pubs (CPIH)


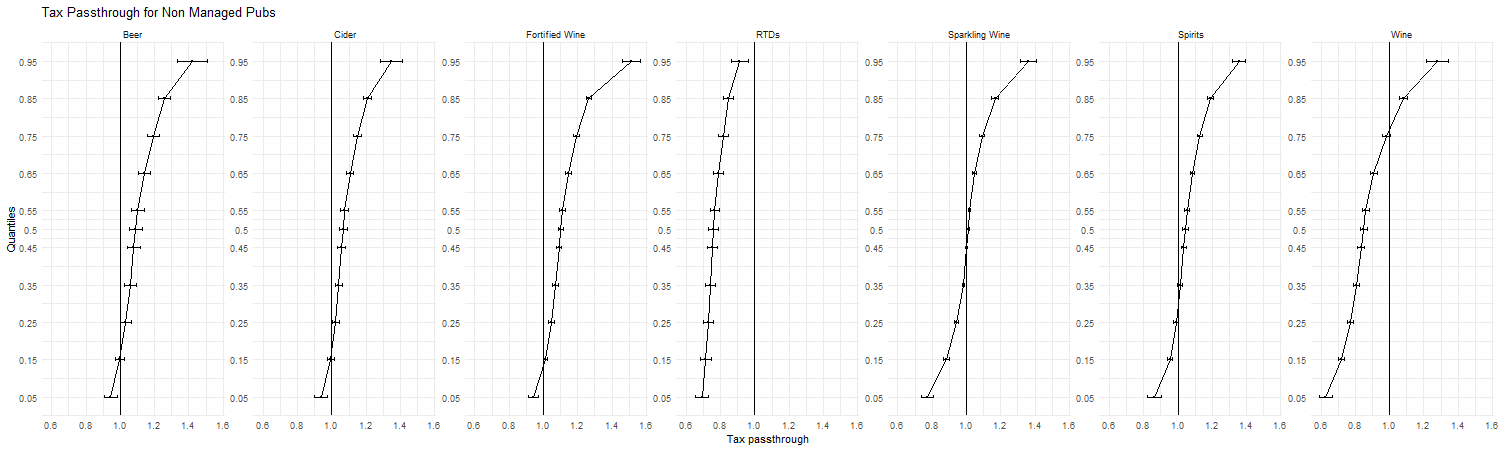


Figure S2d: Managed Pubs (CPIH)


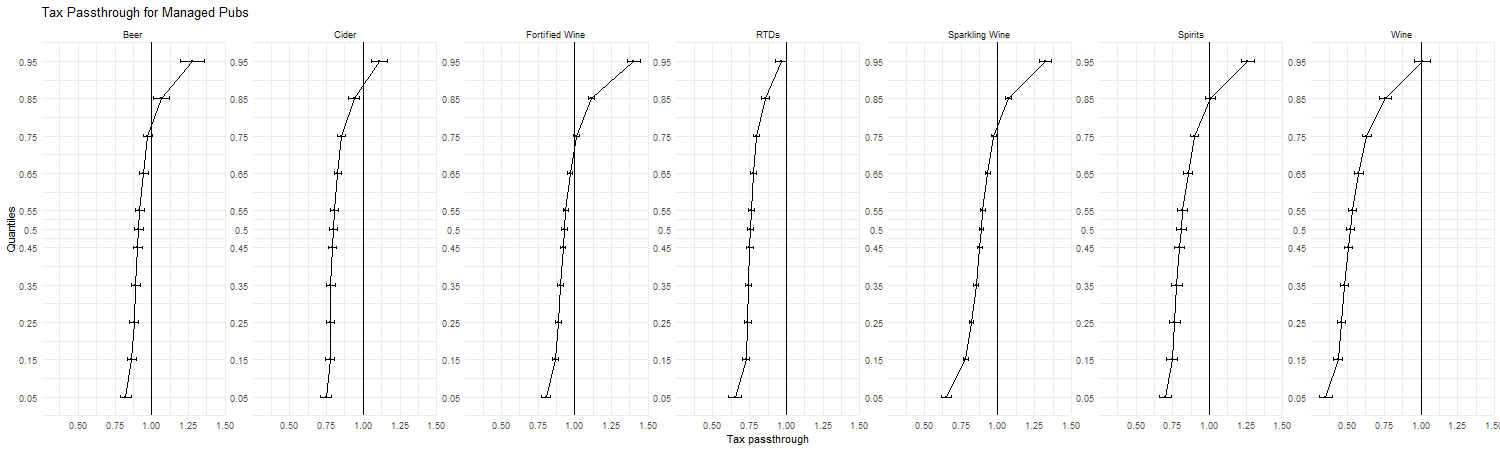


Figure S2e: Restaurants (CPIH)


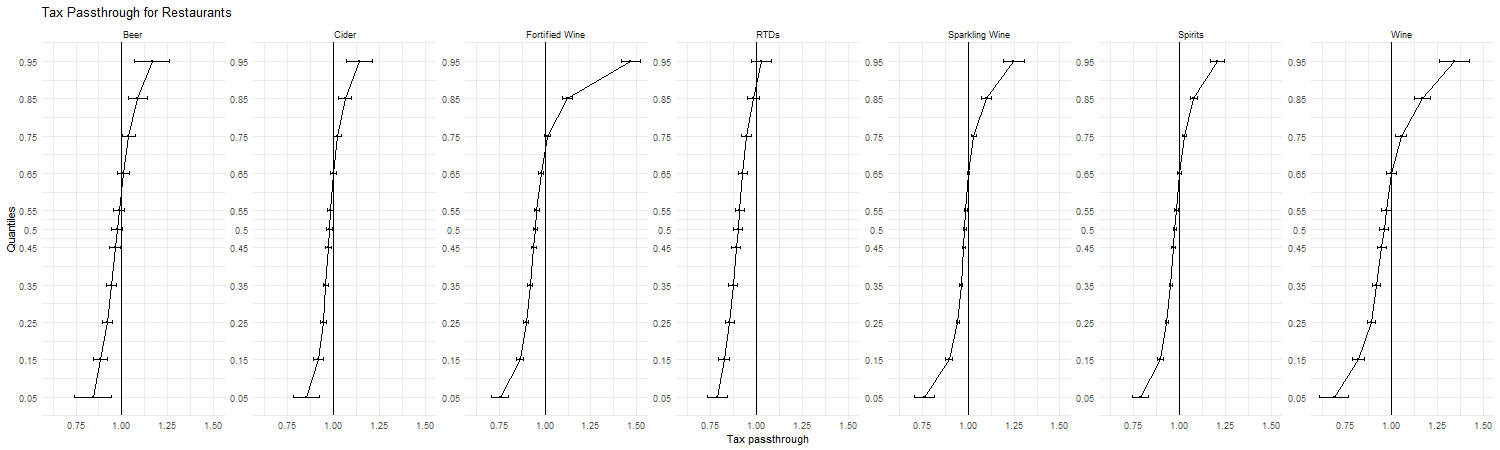


Figure S2f: Hotels (CPIH)


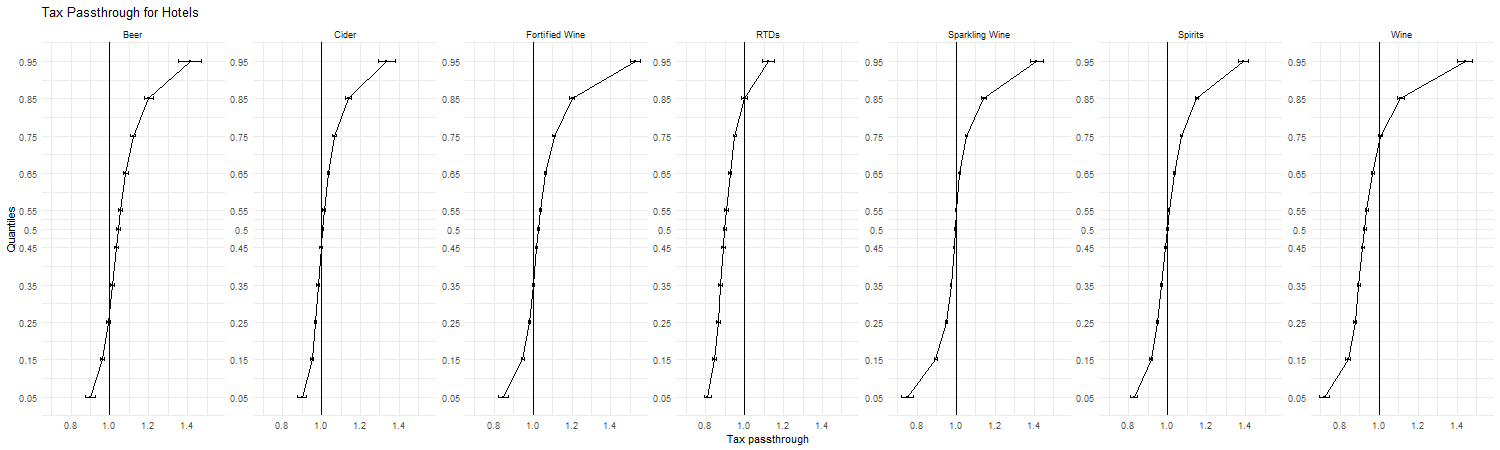


Figure S2h: Proprietary Clubs (CPIH)


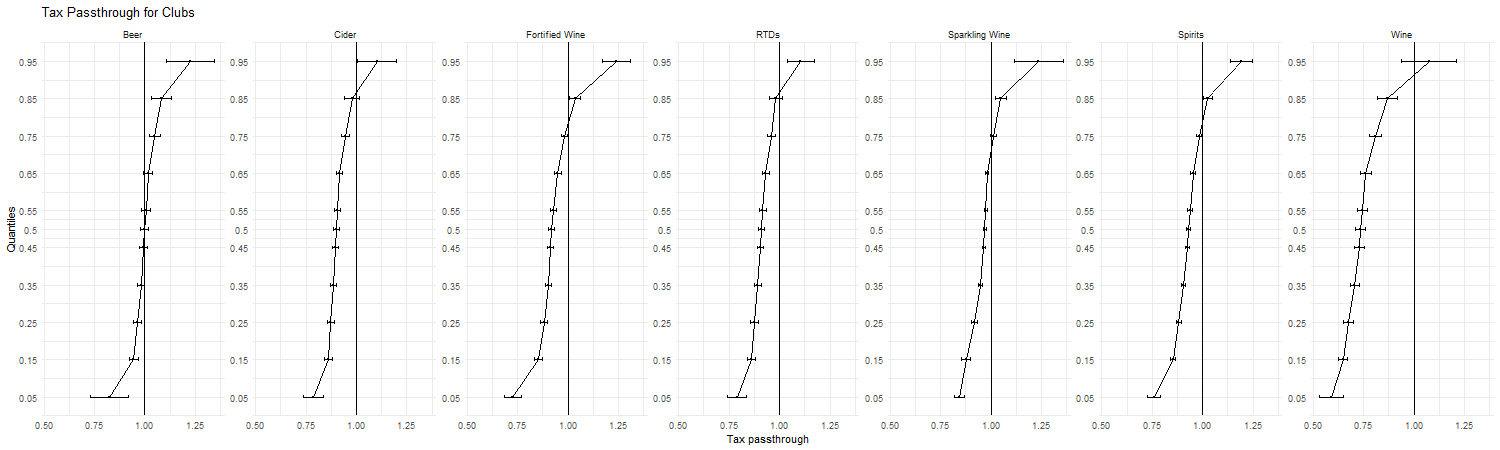


Figure S2i: Sports and Social Clubs (CPIH)


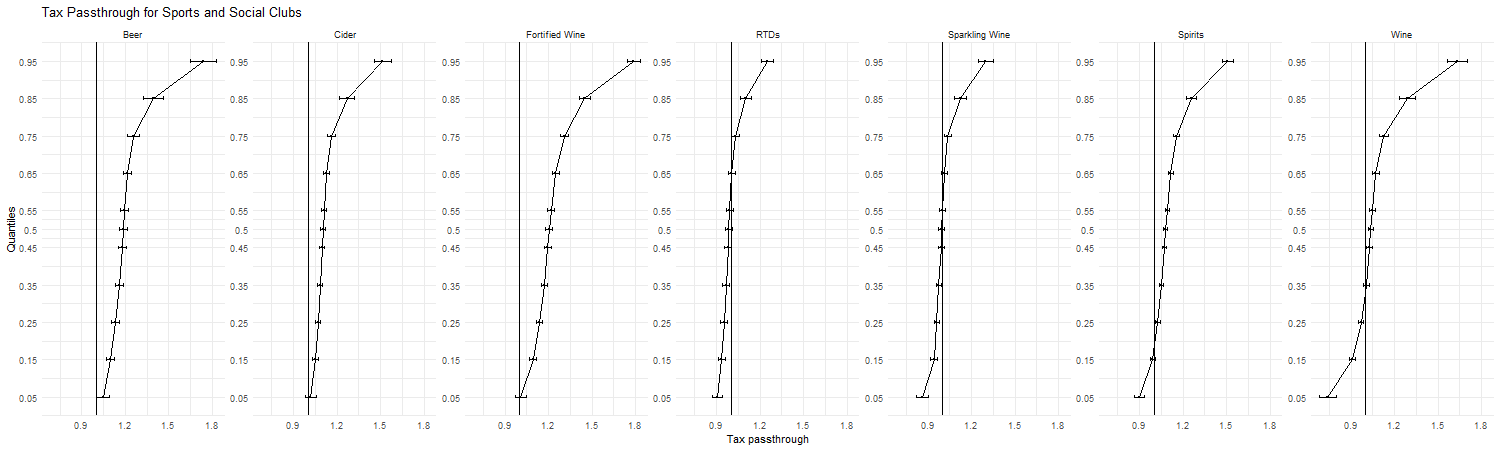


**Figure S3:** Model Estimates by Geographical location in England using RPI

Figure S3a: London


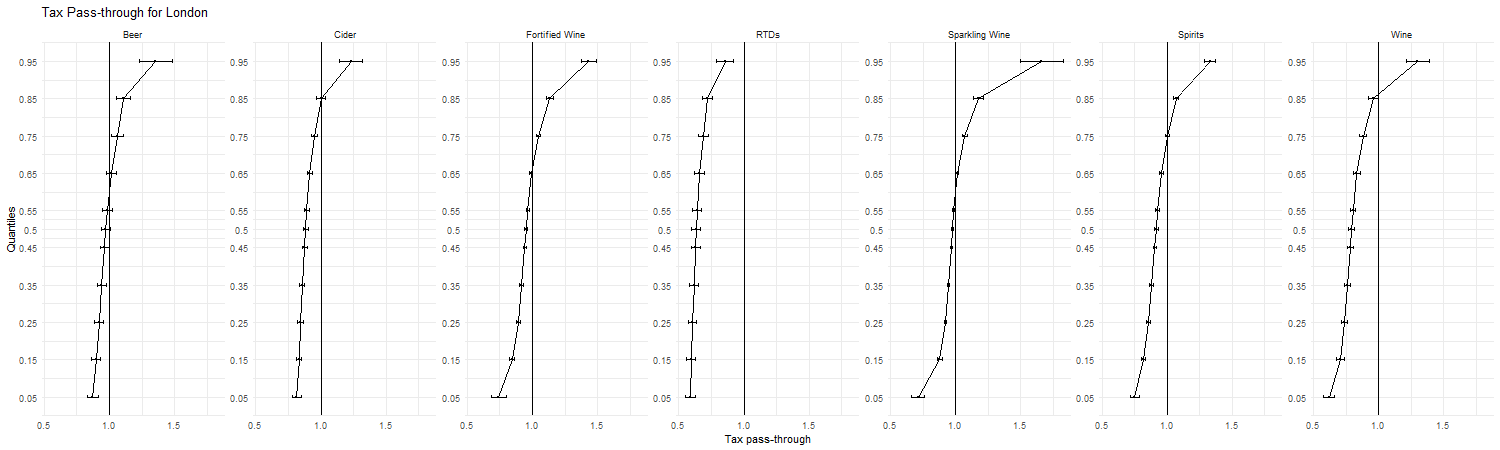


Figure 3b: The Midlands


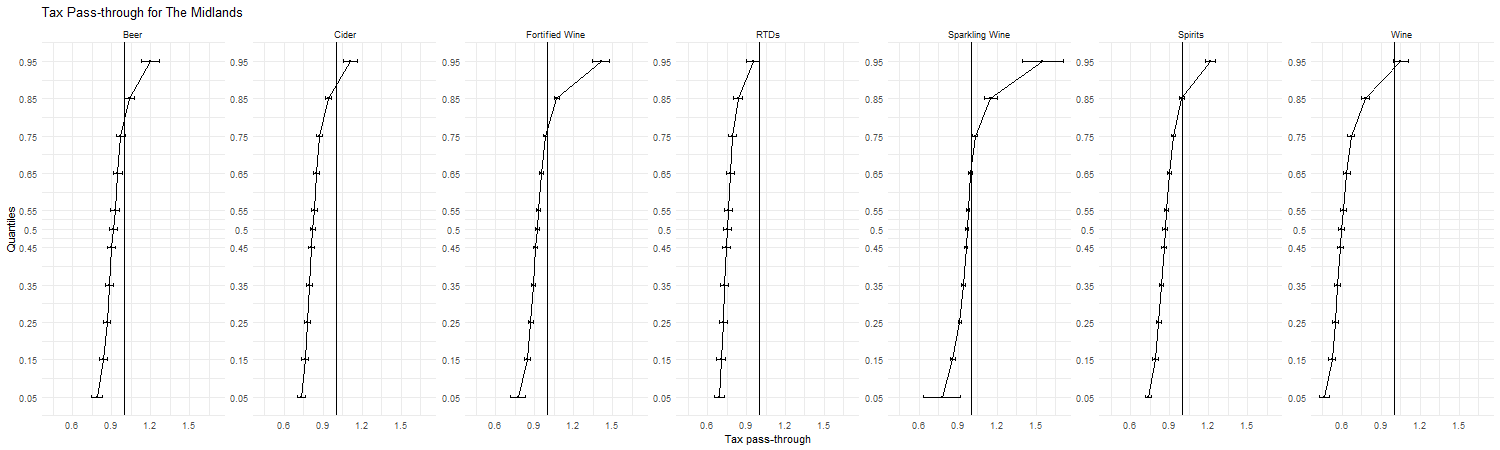


Figure 3c: The North East


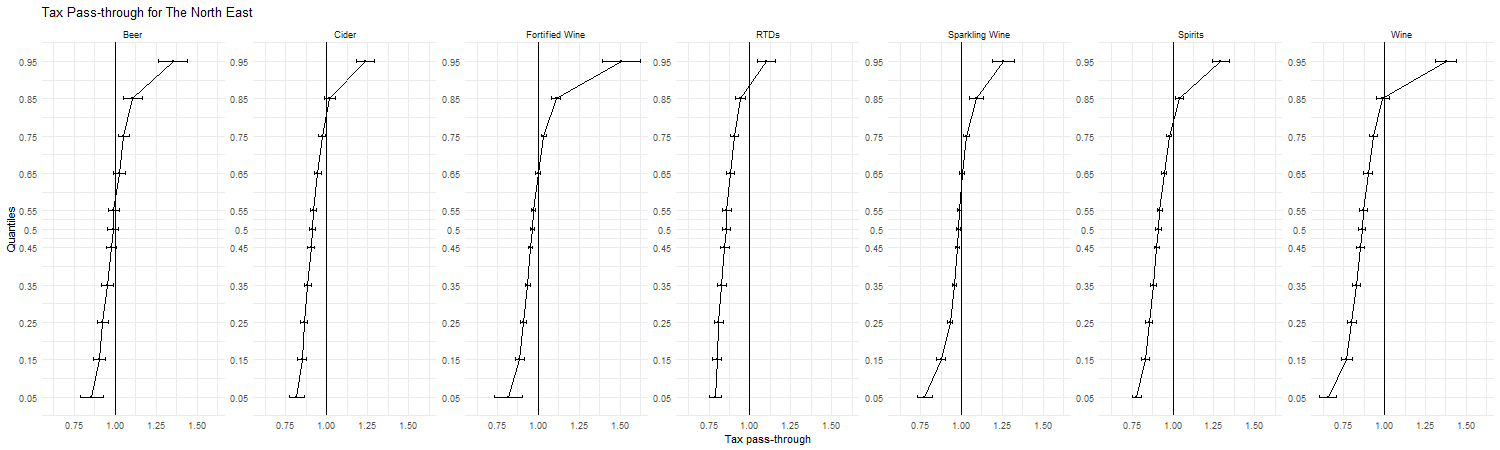


Figure 3d: The South


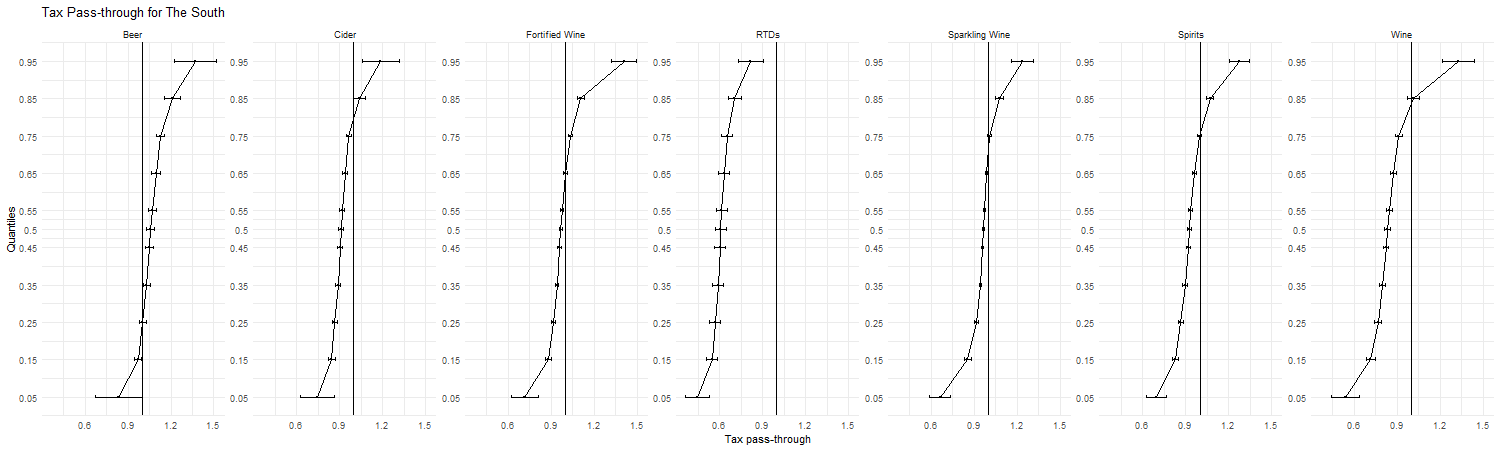


Figure 3e: Yorkshire


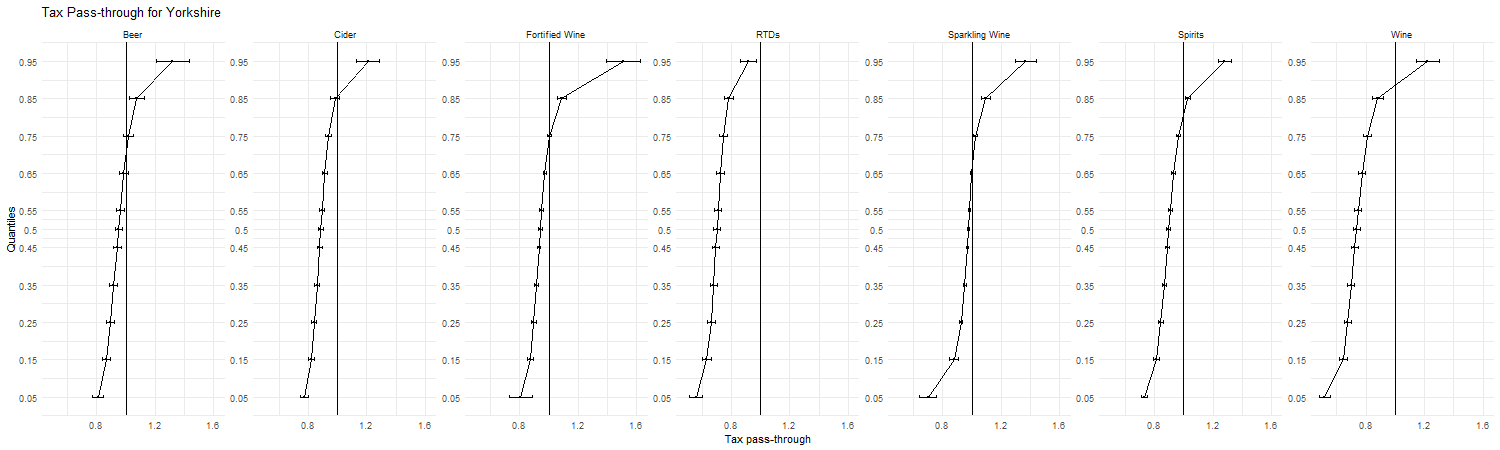


Figure 3f: East Anglia


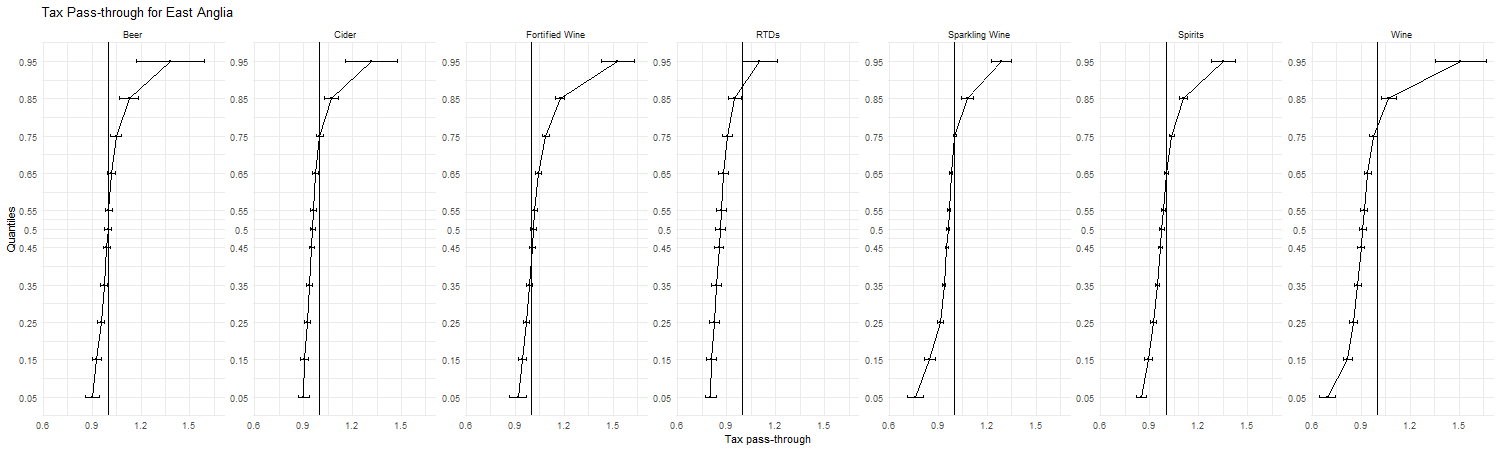


Figure 3g: Harwest England


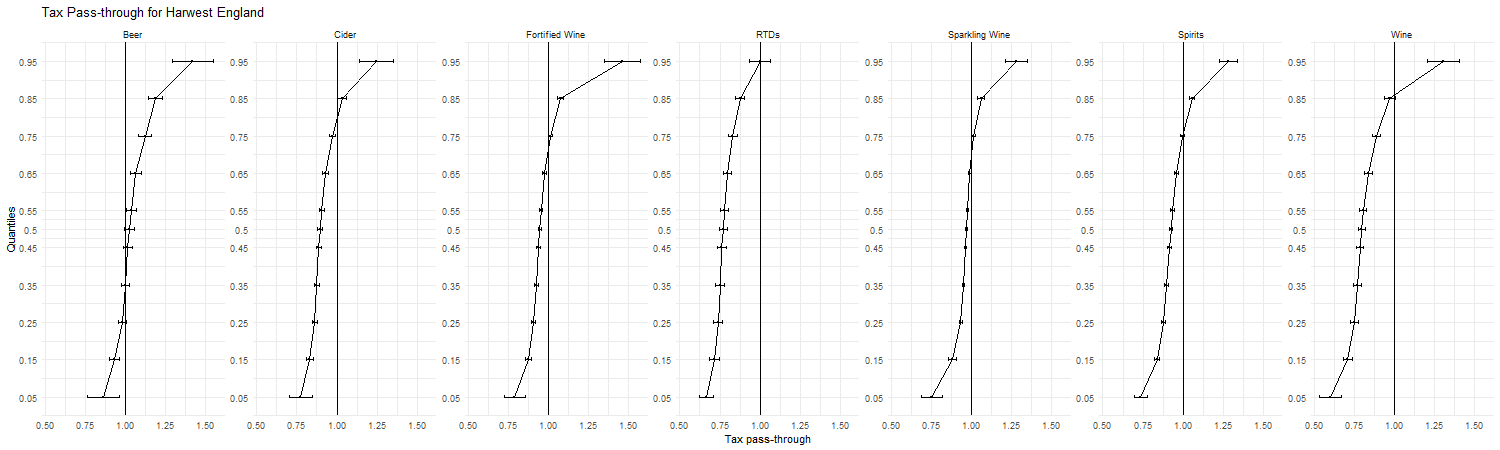


Figure 3h: Lancashire


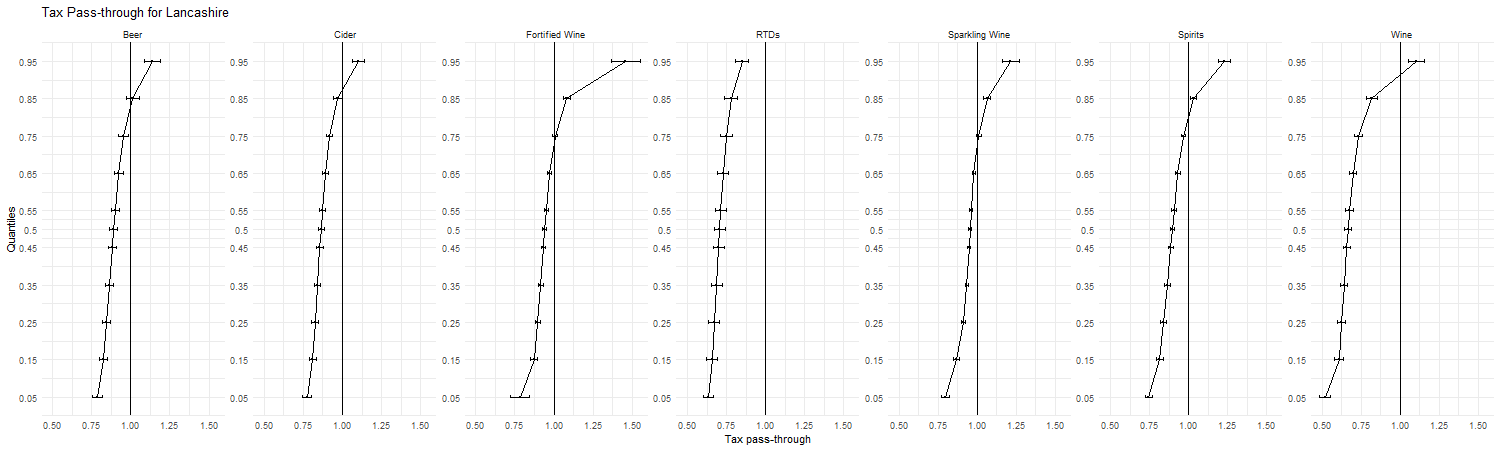


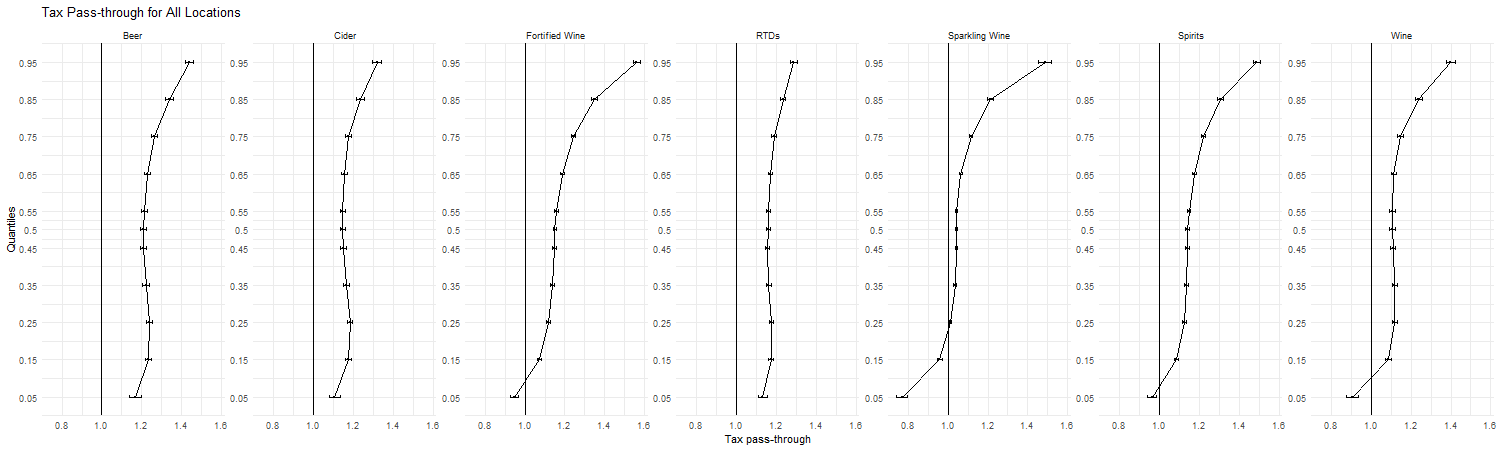
**Figure S4:** Model estimates for all outlet locations excluding inflation in the expected price.

**Figure S5**: Percentage Sold in Each Price Band by Product Type


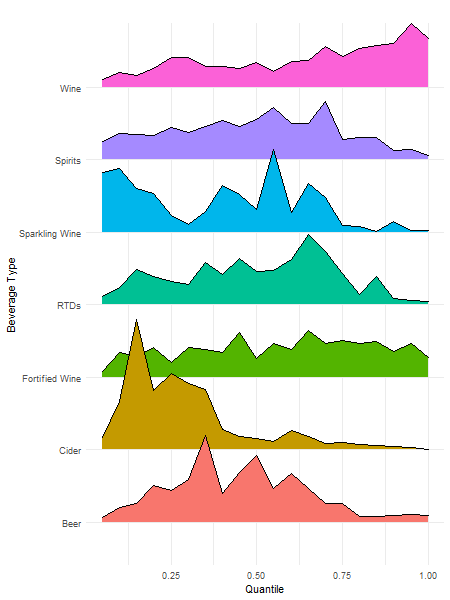


**Figure S6**: Percentage Sold in Each Price Band by Product Type and On-trade Outlet
